# Cerebellar-inspired algorithm for adaptive control of nonlinear dielectric elastomer-based artificial muscle

**DOI:** 10.1098/rsif.2016.0547

**Published:** 2016-09

**Authors:** Emma D. Wilson, Tareq Assaf, Martin J. Pearson, Jonathan M. Rossiter, Sean R. Anderson, John Porrill, Paul Dean

**Affiliations:** 1 Sheffield Robotics, University of Sheffield, Sheffield, UK; 2 Department of Psychology, University of Sheffield, Sheffield, UK; 3 Bristol Robotics Laboratory, University of the West of England and University of Bristol, UK; 4 Department of Engineering Mathematics, University of Bristol, Bristol, UK; 5 Department of Automatic Control and Systems Engineering, University of Sheffield, Sheffield, UK

**Keywords:** cerebellum, artificial muscle, adaptive-inverse control, soft robotics, nonlinear control, transfer of training

## Abstract

Electroactive polymer actuators are important for soft robotics, but can be difficult to control because of compliance, creep and nonlinearities. Because biological control mechanisms have evolved to deal with such problems, we investigated whether a control scheme based on the cerebellum would be useful for controlling a nonlinear dielectric elastomer actuator, a class of artificial muscle. The cerebellum was represented by the adaptive filter model, and acted in parallel with a brainstem, an approximate inverse plant model. The recurrent connections between the two allowed for direct use of sensory error to adjust motor commands. Accurate tracking of a displacement command in the actuator's nonlinear range was achieved by either semi-linear basis functions in the cerebellar model or semi-linear functions in the brainstem corresponding to recruitment in biological muscle. In addition, allowing transfer of training between cerebellum and brainstem as has been observed in the vestibulo-ocular reflex prevented the steady increase in cerebellar output otherwise required to deal with creep. The extensibility and relative simplicity of the cerebellar-based adaptive-inverse control scheme suggests that it is a plausible candidate for controlling this type of actuator. Moreover, its performance highlights important features of biological control, particularly nonlinear basis functions, recruitment and transfer of training.

## 1. Introduction

Making robots ‘soft’ significantly increases the range of environments in which they can operate, allowing them, for example, to interact safely with people (for recent review, see [1]). However, robots made wholly or in part from materials that change the shape when subjected to force are more difficult to control than rigid robots [2].

**Figure 1.**
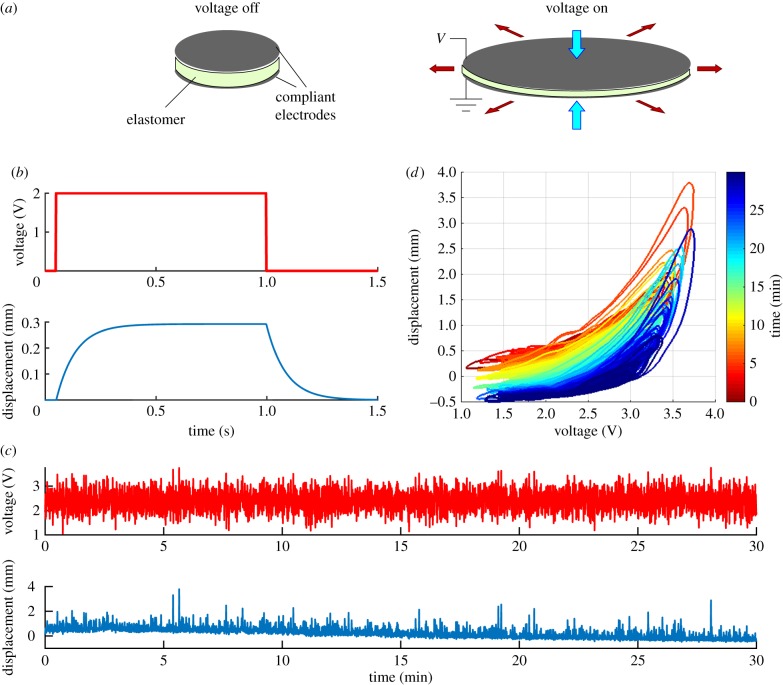
Dielectric elastomer actuators (DEAs) are difficult to control. (*a*) Sketch of DEA operation. Voltage applied to the electrodes produces electrostatic pressure that squeezes and expands the elastomeric film between them. When the voltage is switched off, the film returns to its original shape (cf. [6]). (*b*) Time course of displacement response to a step change in voltage (ordinate shows voltage prior to amplification by a factor of 800). The time course can be approximated by a single exponential, with time course in this case of approximately 100 ms [7]. The responses shown in this and the subsequent panels were obtained from DEAs made of acrylic elastomer (3M VHB 4905) with conductive layers of carbon grease as the electrode plates [7,8] (further details in Methods.). The schematic response shown here is derived from the nonlinear Hammerstein model developed by Wilson *et al*. [7] that accounts for 96–98.8% of the variance in the responses of six DEA samples to filtered white noise. (*c*) The top trace shows the coloured-noise voltage input (prior to amplification, cf. panel *b*) over a 30 min period of stimulation. The bottom trace shows the corresponding displacement response of a DEA sample. The response gradually changes (‘creeps’) over the 30 min period. (*d*) Data from panel *c* replotted to show displacement as a function of voltage for successive time periods as indicated by the colour scale. The displacement response is nonlinear, displays hysteresis, and varies over time (from fig. 1*e* of [8]).

This is true for compliant actuators, capable of muscle-like high strain, which have been manufactured from a wide variety of materials including electroactive polymers (EAPs) [3] that can undergo large deformations in response to electrical stimuli. Dielectric elastomer actuators (DEAs) are an example of compliant EAP-based actuators with high energy density, large strain capability and a relatively fast response [4]. As such, they possess many of the desirable properties of biological muscle [5] and have attracted significant interest in the field of soft robotics research. However, even with recent advances in materials science and manufacturing processes, the precise control of DEAs remains a non-trivial problem owing to a number of intrinsic nonlinear and time variant characteristics as illustrated schematically in figure 1.

When a membrane of elastomer is sandwiched between two compliant electrodes, applying a voltage to the electrodes causes the membrane to flatten and expand (figure 1*a*). A typical time course for this response to step changes in voltage is shown in figure 1*b*, where steady state is reached only after a substantial delay (in this case, approx. 300 ms). With a coloured-noise voltage input delivered for 30 s, the displacement response gradually changes (figure 1*c*). When these data are plotted as voltage versus displacement at different time points (figure 1*d*), it can also be seen that the response is a nonlinear function of input voltage and shows hysteresis, as well as increasing in amplitude with time (figure 1*d*). Furthermore, not shown in the figure, significant effort is required in the manufacturing process of DEAs to reduce variance in the response between individual actuators; they are sensitive to temperature; and, when loaded, prone to failure and, for acrylic elastomers, systematic degradation over time. These issues and phenomena are apparent in both dielectric- and ionic EAP-based actuators [3,9] and constitute one of the main challenges to overcome before the technology can be incorporated more broadly into robotic systems. There is ongoing research into improving the material properties of DEAs, such as by using silicone, to address these challenges. However, this research focuses on control.

The similarities between DEAs and biological muscles referred to above extend to these control problems, which also characterize biological muscles. The question therefore arises of whether biological control strategies, which have evolved to deal with compliant materials, might show promise for the control of DEA-based actuators. These strategies are probably best understood for the extraocular muscles (EOMs) that control the eye, because for these muscles, the poorly understood effects of proprioception are less prominent than for skeletal muscles, and their neural control machinery does not involve the very complex organization of the spinal cord [10]. In broad terms, it appears that eye-movement-related neurons in the brainstem implement an approximate inverse model of the oculomotor plant, i.e. the EOMs and orbital tissue [11,12]. This approximate model is calibrated by the cerebellum, which is thought to ensure eye-movement accuracy by using a form of supervised learning, in which information about movement inaccuracy adjusts weights in a specialized neural network [13]. The combination of brainstem model and continual cerebellar calibration appears able to cope with the kinds of control problems illustrated in figure 1, as manifested by the oculomotor plant.

**Figure 2.**
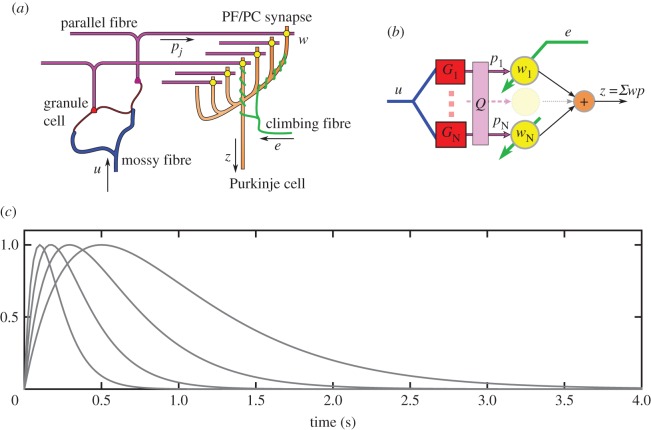
(*a*) Cerebellar microcircuit as an adaptive filter. (*a*) Highly simplified diagram of cerebellar cortical microcircuit. Details in text. Not shown are Golgi cells, which receive input from mossy and parallel fibres and send inhibitory projections back to the synapses between mossy fibres and granule cells. This recurrent inhibitory network contributes to the recoding of mossy fibre inputs by granule cells (Discussion). (*b*) Interpretation of cerebellar microcircuit as an adaptive linear filter. Details in text. (*c*) Alpha function basis. Normalized impulse responses of alpha basis functions. (Online version in colour.)

**Figure 3.**
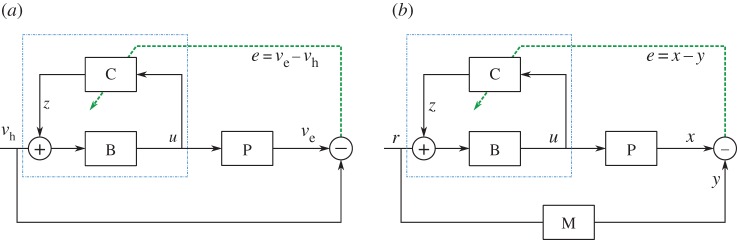
Basic architecture for motor plant compensation. (*a*) Linearized model of the horizontal VOR, the reflex that stabilizes images on the retina by reducing retinal slip. The vestibular system (not shown) generates a head velocity signal v_h_. Retinal slip (error, *e*) is zero when the eye velocity *v*_e_ exactly opposes the head velocity *v*_h_. Control of the oculomotor plant (P) is provided by a combination of a brainstem filter (B) and recurrently connected adaptive cerebellar filter (C). (*b*) Architecture for position control of a nonlinear DEA plant using a control scheme based on the VOR. Here, compensation is again provided by a combination of B and C; however, the position as opposed to velocity is controlled, a reference model (M) is included such that a filtered version of the reference input is tracked, and the elements represented in the diagram are not necessary linear filters. (Online version in colour.)

We therefore investigated how far a similar scheme could be used to control DEA [7] by employing a modified version of a simplified model of the cerebellum and brainstem circuitry, previously developed in the context of oculomotor plant compensation [14,15]. In this model (figures 2 and 3: details in following sections), the cerebellum is represented by an adaptive filter [16,17] whose input is an efference copy of the commands sent to the plant. A measure of movement inaccuracy (retinal slip in the case of the oculomotor system) is sent to the adaptive filter as an error signal. The standard least mean square (LMS) learning rule is then used to adjust the adaptive-filter weights, so that the error is reduced, an example of adaptive-inverse control [18]. Application of this recurrent-architecture scheme to DEAs within their linear range of operation (figure 1*d*) produced accurate control of displacement despite variation in dynamics between actuators, and within an actuator as a function of time (figure 1*c,d*).

Here, we seek to extend these findings to the nonlinear range of DEA operation (figure 1*d*), by altering the linear model in three ways. First, the adaptive filter model is expanded to allow it to produce nonlinear outputs, using a thresholding scheme similar to that described by Spanne & Jörntell [19] which is based on the properties of neural processing in the granular layer of the cerebellum. Second, the brainstem model is also expanded to allow the production of nonlinear outputs, in this case by mimicking the effects of recruitment. Biological muscles are composed of motor units arranged in parallel, with each unit controlled by its own motoneuron (for most muscles). To increase the force exerted by the muscle, the control signal sent to the motoneuron pool changes its firing in two ways. One is an increase in the number of motoneurons firing (recruitment), the other is an increase in the firing rate of those motoneurons already recruited [20]. Because later recruited units are typically more powerful than those with lower thresholds for both skeletal muscles [21] and probably EOMs [22], a nonlinearity of the kind shown in figure 1*d* could, in principle, be accommodated by appropriate recruitment. Finally, an additional learning mechanism is introduced that allows cerebellar output to ‘teach’ the brainstem, thereby allowing the transfer of large gains from the cerebellum to the brainstem. Transfer of this kind has been observed in the oculomotor system (references in [23]).

Evaluating this bioinspired control scheme for DEAs has implications not only for the control of DEA-based actuators, but also for understanding cerebellar function. Webb [24] explains the general usefulness of robotics for clarifying and evaluating hypotheses in neuroscience: here, the specific hypotheses concern the competencies of the adaptive filter model of the cerebellum and the recurrent architecture for the control of compliant actuators.

The paper is structured as follows. Methods section describes first the components of the algorithm that is the adaptive filter model of the cerebellar microcircuit and the recurrent architecture for plant compensation. It then outlines the changes made to the algorithm to deal with DEA nonlinearities, resulting in three new control schemes, and in the final section describes the experimental set-up. The Results section shows the effects of applying the new control schemes compared with conventional PID control, and the Discussion section considers their limits and significance. Finally, appendix A provides the mathematical details of the control algorithms.

## 2. Methods

### 2.1. Cerebellum: the adaptive filter model

The cerebellar cortical microcircuit can be modelled as an adaptive filter [16,17]. The main features of the microcircuit are shown schematically in figure 2*a*, and translated into adaptive-filter form in figure 2*b*. In this model, the main cerebellar inputs carried by mossy fibres (figure 2*a*) are represented by *u*. These are recoded by a bank of fixed filters *G_1_* *…* *G_N_* corresponding to processing in the granular layer, giving rise to outputs *p_1_* *…* *p_N_* that correspond to signals in parallel-fibres. The parallel-fibre signals are weighted (*w_1_* *…* *w_N_*, corresponding to synapses between parallel fibres and Purkinje cells) and summed linearly (by Purkinje cells) to give the filter output *z*. The Purkinje cells also receive input via a single climbing fibre. This input acts as a teaching signal (in the simulations presented here the teaching signal is the tracking error *e*, that is the difference between actual and desired actuator position). The Purkinje cell synaptic weights are modified over time according to the covariance learning rule 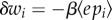, which corresponds to the LMS learning rule [25].

Much of the power of the adaptive filter depends on how far the basis filters *G*_1_, … , *G_n_* provide a rich recoding of the input, allowing synthesis of a large range of desired outputs. In engineering applications, the basis is often taken to be a bank of tapped delay lines. However, a very large number of delay lines may be required to represent the long time-constant behaviours characteristic of biological systems. We therefore use an alternative basis better adapted to biological control, namely a set of alpha functions [7] in which the average delay increases logarithmically (figure 2*c*). These cover a large range of time constants very economically, although filter width increases proportionally to delay giving less accurate time-location at increasing delay.

Both log-spaced alpha functions (and tapped delay lines) have highly correlated outputs that drastically affect the speed of learning. For learning rates to be maximized, the basis filter outputs must be mutually uncorrelated and have equal power [26]. It is thought that unsupervised plasticity mechanisms within the granular layer may reduce correlations between granule cell outputs [27]. We model these decorrelation processes by applying a further processing stage to the filter outputs, represented by the unmixing matrix *Q* in figure 2*b*. This matrix is estimated using singular value decomposition based on a batch of filter outputs to provide uncorrelated, unit power, parallel fibre signals [7].

Although the cerebellum is involved in a very wide variety of tasks, the microcircuit itself is relatively homogeneous over the entire cortex [13]. This implies that the same adaptive filter model underlies many different processing tasks, so a fundamental design rule for our biomimetic control scheme is that the basic filter design should not be modified in *ad hoc* ways for different control applications. Instead, task-specific processing is obtained by embedding the adaptive filter in a range of different connectivities [12].

### 2.2. Recurrent architecture

In the linear case embedding, the cerebellar learning element in a recurrent architecture (figure 3*a*) simplifies the adaptive control problem [14,15]. In this architecture, inspired by the organization of the cerebellar flocculus and oculomotor brainstem to maintain stability of eye gaze, referred to as the vestibulo-ocular reflex (VOR), the controller has two main parts.

(1) The fixed brainstem part of the controller **B** converts a signal representing head velocity *v*_h_ into a control signal *u* which is sent to the oculomotor plant **P**. In the VOR, the task is to move the eyes in the opposite direction to the head, so that eye velocity *v*_e_ is equal to −*v*_h_, thereby stabilizing the image on the retina. The brainstem constitutes an approximate inverse of the plant (P^−1^).
(2) The adaptive part of the controller **C** receives an efferent copy of the motor commands *u* generated by the brainstem. If these commands are inaccurate, then the resultant eye movements will not match the head movements, and the image will move across the retina generating a retinal slip-error signal *e*. This signal drives learning in **C**, which adjusts its output *z* to the brainstem so as to reduce *e*. When learning is complete the combined controller approximates the inverse of the plant transfer function [18], and the cerebellum has learnt an incremental plant model **C** = **B^−1^** – **P**.

An important feature of the recurrent architecture shown in figure 3*a* is that it can use sensory errors to drive adaptation directly, rather than needing to estimate what the required motor command should have been [12,28]. In particular, it guarantees that the teaching signal required for stability and convergence is simply the tracking error rather than a more complex teaching signal [15].

Figure 3*b* shows how the basic recurrent architecture was altered for control of a DEA in its linear operating range, using a biohybrid approach that incorporates model reference control [7]. After learning, the behaviour of the controlled plant matches that of the reference model **M** (i.e. it tracks *y* which is a filtered version of *r*) which specifies a realistic response for the controlled plant; the use of a reference model also ensures that the estimated controller is proper. Using model reference, adaptive control is a technical solution that enables the cerebellar algorithm to function independently of the plant order.

### 2.3. Dealing with nonlinearity

**Figure 4.**
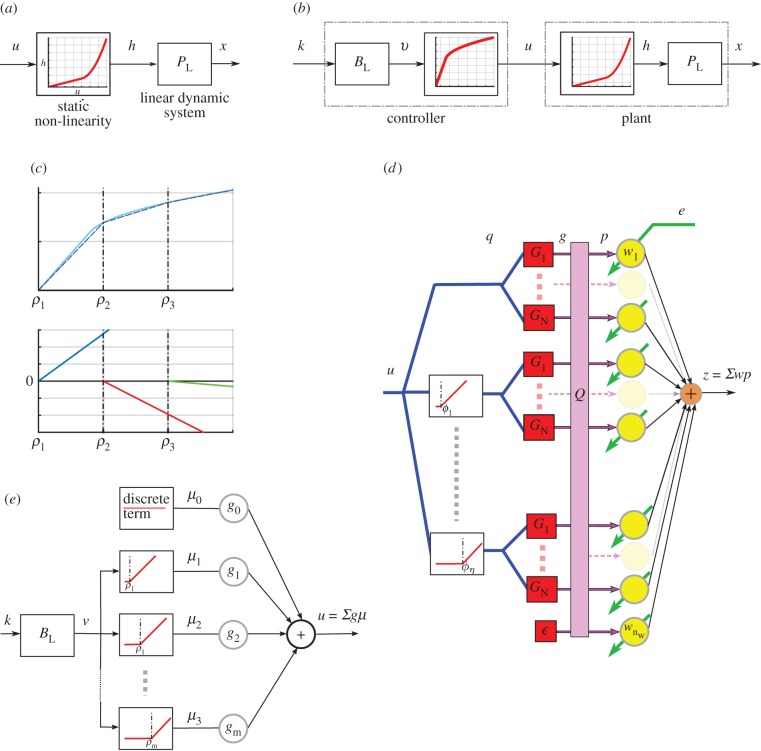
Nonlinear inverse control. (*a*) General Hammerstein model of a system. (*b*) Pictorial representation of perfect compensation of the Hammerstein system. (*c*) Demonstration of how piecewise linear elements can be used to construct a nonlinear function. (*d*) Nonlinear cerebellar adaptive filter, as an extension of the adaptive linear filter shown in figure 2*b*. (*e*) Nonlinear brainstem used to control the DEA. Details for (*c–e*) are given in appendix A. (Online version in colour.)

Nonlinear plants do not have transfer functions, but the same concept of plant compensation (inverse control) holds if the plant has an inverse that is stable [29]. We assume here that the DEA plant has an inverse that is stable (i.e. bounded output implies bounded plant input), a reasonable assumption given that the input signal must always be kept small enough to avoid damage. For the DEAs used in this study, the plant can be represented by a Hammerstein model [7], that is as a static nonlinearity (SNL) followed by a linear dynamic system (LDS; figure 4*a*). Such a plant can be perfectly compensated if the controller contains an LDS equal to the inverse of the plant LDS followed by an SNL equal to the inverse of the plant SNL (figure 4*b*).

Here, we use a series of piecewise linear elements to approximate the continuous nonlinear function that constitutes the SNL, as shown figure 4*c* (equation (A 9) in appendix A). Two methods were tried, both of them bioinspired and consistent with the basic circuitry of the adaptive filter and the recurrent architecture.

(1) One of the features of recurrent inhibition in the granular layer is that it can provide a natural thresholding mechanism for granule cell responses. Spanne & Jörntell [19] have argued that the resulting threshold-linear processing elements may be useful for nonlinear control problems. We therefore incorporated a bank of threshold-linear elements with varying threshold as a pre-processing stage (see figure 4*d* and equations (A 6) and (A 7) in appendix A) providing a flexible set of nonlinear basis filters.
(2) Threshold nonlinear elements are also found in the brainstem. Oculomotor neurons have a wide range of thresholds [30], and it has been suggested that recruitment can be used to linearize nonlinear plants [31]. We therefore investigated whether a bank of threshold linear units in the brainstem (figure 4*e*) could compensate for the DEA plant nonlinearity.

The final control scheme to be examined included an additional site of plasticity in the brainstem (equation (A 10) in appendix A), inspired by the existence of such a site in the vestibular nuclei that allows the cerebellar input to drive brainstem learning during VOR adaptation [32]. This mechanism can be used to transfer models learnt in the cerebellum to the brainstem [23], and predicts a heterosynaptic learning rule using correlations between the brainstem input and the inhibitory cerebellar input drive that has been verified experimentally [33]. An advantage of learning transfer is that it limits the amount of gain that is required to be stored in the cerebellar loop, improving loop stability if the plant is subject to large changes over time.

### 2.4. Experimental set-up

**Figure 5.**
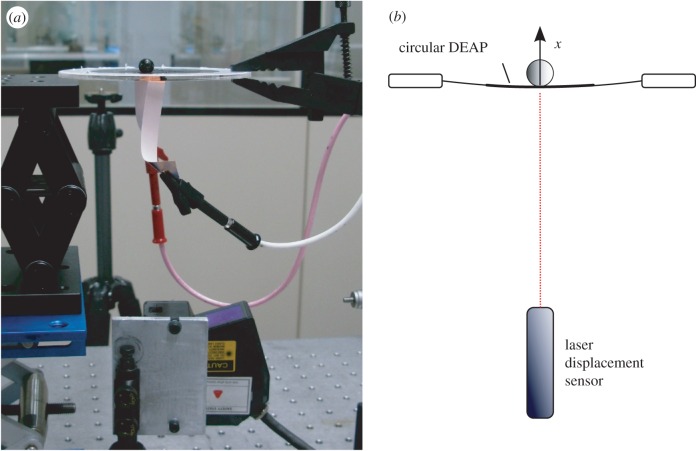
Experimental set-up. (*a*) Photograph of experimental set-up for measuring the vertical displacement of a DEA stretched on a circular Perspex frame supporting a spherical load, using a laser displacement sensor. (*b*) Diagram of the experimental set-up, showing displacement *x*. (Adapted from fig. 2*a* and *b* of [7].) (Online version in colour.)

The experimental set-up was the same as that described previously in Wilson *et al*. [7]. The control task was to drive the 1 degree of freedom displacement response of the DEA to track a filtered coloured-noise reference signal *y* such that the controlled actuator behaved as specified by the reference model **M** (figure 3*b*). Each DEA consisted of a thin, passive elastomeric film, sandwiched between two compliant electrodes (figure 5*a*). Voltage applied to the electrodes squeezed the film and expanded it biaxially. To constrain the controlled variable to 1 degree of freedom, a spherical load was placed at the centre of a circular DEA and its motion in the vertical plane (i.e. vertical displacement) was measured (figure 5*a*,*b*).

The DEAs were made of acrylic elastomer (3M VHB 4905) with an initial thickness of 0.5 mm. This material was chosen owing to its low cost, availability, robustness and adhesive properties that were exploited in the assembly process. The elastomer was pre-stretched biaxially by 350% (where 100% was the unstretched length) to a thickness of approximately 41 µm (unmeasured) prior to being fixed on a rigid Perspex frame with inner and outer diameters of 80 and 120 mm, respectively. A conductive layer of carbon grease (MG chemicals) formed the electrodes that were brushed on both sides of the VHB membrane as circles with a diameter of approximately 35 mm. The load used during experiments was a sphere weighing 3 g.

The control algorithm (table 1) was implemented in LabVIEW and from there embodied in a CompactRio (CRIO-9014, National Instruments) platform, with input module NI-9144 (National Instruments) and output module NI-9264 (National Instruments) used in combination with a host laptop computer. LabVIEW was run on the host laptop computer, with communication between the host laptop and CompactRio (CRio) carried out, using the LabVIEW shared variable engine. In all experiments, all signals were sampled simultaneously with a sampling frequency of 50 Hz.

**Table 1.** Plant compensation control algorithm. Algorithm used to control the response of a DEA. The timing was done using a National Instruments Compact Rio with labVIEW software. Read/write used a read-write National Instruments FPGA module (see Methods). The delay between steps 8–9 was 0.0001 s.

	control algorithm	for each time step, *k*
1	*y_k_* = *M*(*q*, *τ*)*r_k_*	filter input signal through reference model
2	**q***_k_* = *f*_2_(*u_k_*−1)	nonlinear transformation of (previous) motor command
3	**for** *i* = 1 : *n_f_* **do** *g_i,k_* = *G_i_*(*q*, *T_i_*)**q***_k_* **end for**	filter transformed motor commands through bank of alpha filters
4	**p***_k_* = *Q***g***_k_*	transform filter outputs into a faster learning basis
5	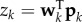	calculate adaptive filter output
6	*v_k_* = *B_L_*(*q*, *γ*)(*r_k_* + *z_k_*)	filter adaptive filter output and input signal through linear brainstem filter
7	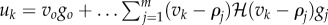	calculate output of piecewise linear, nonlinear brainstem element
8	WRITE *u_k_*	use motor command to drive DEAP
9	READ *x_k_*	measure response of DEAP
10	*e_k_* = *x_k_*−*y_k_*	calculate error between desired and actual response
11	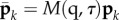	filter parallel fibre signals through reference model
12	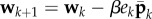	update adaptive filter weights
14	**for** *j* = 1 : *m* **do** if *j* < 2 *g_j_*_,*k*__+__1_ = *g_j_*_,*k*_ + ζ*z_k_μ_j,k_* otherwise *g_j,k_* *_+_* *_1_* = *g_j,k_* + ζ*μ_k_v_j,k_*−ζ*z_k_μ_j−1,k_* **end for**	update gains of piecewise linear brainstem element

A laser displacement sensor (Keyence LK-G152, repeatability—0.02 mm) was used to measure the vertical movement of the mass sitting on the circular DEA. This signal was supplied to the input module of the CRio. From the output module of the CRio, voltages were passed through a potentiometer (HA-151A HD Hokuto Denko) and amplified (EMCO F-121 high-voltage module) with a ratio of 15 V : 12 kV and applied to the DEA.

### 2.5. Control schemes

Six control schemes were applied to the DEA shown in figure 5. In each case, the actuator was required to track for 900 s a low-pass filtered (1 Hz cut-off) white-noise voltage input, with a range of desired displacement amplitudes of 0.1–1.8 mm. This amplitude range corresponds to average motor commands (voltage inputs to the DEA) of the order of 3 V prior to amplification. These inputs excite the full nonlinear dynamics of the DEA.

Five schemes used a model brainstem and recurrently connected cerebellar adaptive filter to compensate for the DEA dynamics, an arrangement previously suggested for compensation of the oculomotor plant in animals and humans. All were tested in simulation, and the fifth also applied experimentally. In addition, a PID-based control scheme was tested in simulation for comparison.

## 3. Results

**Figure 6.**
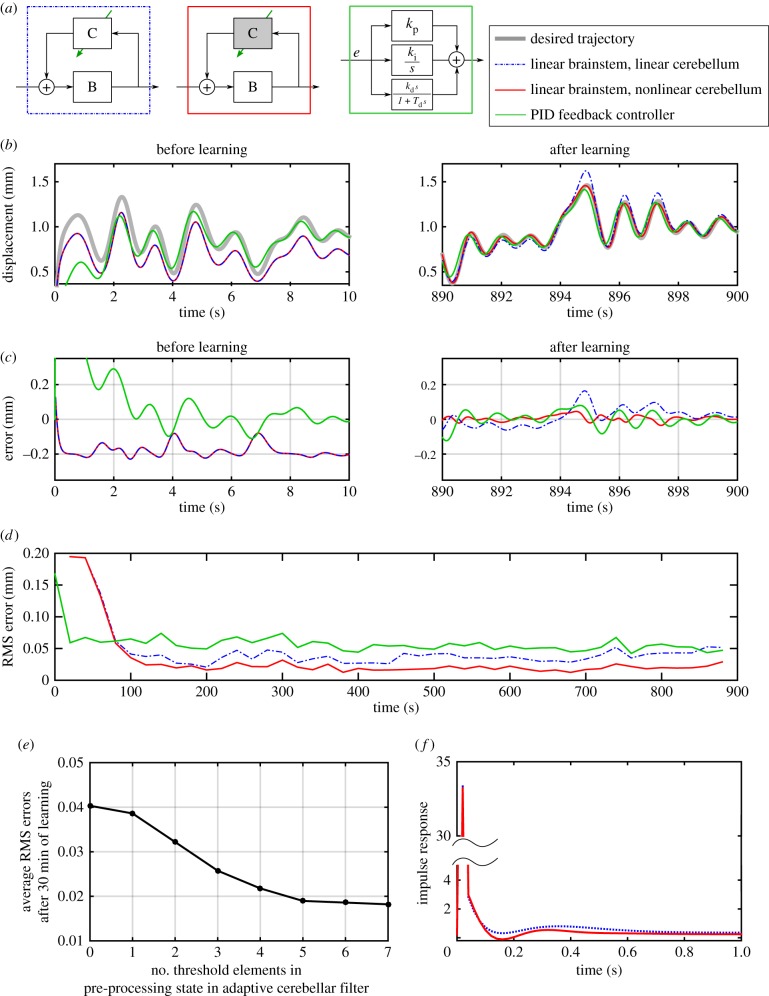
Linear versus nonlinear cerebellar control. Simulated results for DEA control using three different schemes. (*a*) Diagram of the three control schemes. An arrow indicates an adaptive element, and a shaded box a nonlinear element. (*b*) Tracking a desired displacement signal using each controller. The left-hand panel shows the desired and actual responses before learning for the two cerebellar-based schemes, and the right hand, the response after learning compared with the response of the PID controller. (*c*) Errors in displacement tracking corresponding to tracking response shown in panel *b*. (*d*) Windowed RMS errors during learning for each controller. Errors are smallest using the nonlinear cerebellum-based controller. (*e*) RMS errors averaged over final 320 s of 30 min of learning shown for the nonlinear controller as a function of the number of nonlinear elements. (*f*) Response of the two learned cerebellum–brainstem controllers to an impulse input. The response is the output when a pulse of length d*t* (sample time—0.02 s) and magnitude 1/d*t* was input to the learned controller.

The first control scheme applied to the DEA (see Methods) used the linear brainstem and cerebellar models (figure 6*a*) previously applied to both simulated and experimental control of the DEA in its linear range [7]. The performance of the fixed linear brainstem (defined in table 2) before and after learning is shown in figure 6*b*,*c*. As expected, the linear control scheme cannot fully compensate for the nonlinear plant dynamics, having particular trouble tracking larger peaks in the desired displacement response. Its use, here as a reference condition, gives an indication of the problems caused by the nonlinearity, with its steady-state RMS error (figure 6*d*) being 0.04 mm. For comparison, the linear control scheme gives steady-state RMS errors of 0.011 mm when the DEA is excited over a reduced range (i.e. reference signal reduced to a maximum of 1 mm), such that the dynamics can be approximated as linear [7]).

**Table 2.** Parameters for experiments. Parameters used to control the response of a DEA. The third experiment was linear PID control for which control parameters are provided in appendix A.

parameter	experiment	value
number of piecewise linear brainstem terms	first	*m* = 1
	second	*m* = 1
	fourth	*m* = 8
	fifth	*m* = 8
	sixth	*m* = 8
thresholds for brainstem piecewise linear terms	first	*ρ*_1_ = 0
	second	*ρ*_1_ = 0
	fourth	*ρ*_1−8_ = [0 0.255 0.51 0.765 1.02 1.275 1.53 1.785]
	fifth	*ρ*_1−8_ = [0 0.255 0.51 0.765 1.02 1.275 1.53 1.785]
	sixth	*ρ*_1−8_ = [0 0.255 0.51 0.765 1.02 1.275 1.53 1.785]
initial brainstem gains	first	*g*_0−1_ = [2.1 0.9]
	second	*g*_0−1_ = [2.1 0.9]
	fourth	*g*_0−8_ = [0.92 2.38 1.07 −1.92 −0.78 −0.11 −0.12 −0.045 0]
	fifth	*g*_0−8_ = [0.92 2.38 1.07 −1.92 −0.78 −0.11 −0.12 −0.045 0]
	sixth	*g*_0−1_ = [2.1 0.9 0 0 0 0 0 0 0]
rate of learning brainstem gains	first	*ζ* = 0
	second	*ζ* = 0
	fourth	*ζ* = 0
	fifth	*ζ* = 0
	sixth	*ζ* = 0.01
number of nonlinear cerebellar elements	first	*ν* = 0
	second	*ν* = 5
	fourth	*ν* = 0
	fifth	*ν* = 5
	sixth	*ν* = 5
thresholds for nonlinear cerebellar elements	first	n.a.
	second	*σ*_1−5_ = [2.18 2.48 2.78 3.08 3.38]
	fourth	n.a.
	fifth	*σ*_1−5_ = [2.18 2.48 2.78 3.08 3.38]
	sixth	*σ*_1−5_ = [2.18 2.48 2.78 3.08 3.38]
discrete alpha basis filters	all	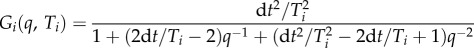
number of alpha filters	all	*n_f_* = 4
time constants of alpha filters	all	log-spaced from *T*_1_ = 0.1 to *T*_4_ = 0.5
fixed cerebellar bias	all	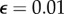
rate of error learning	all	*β* = 8
discrete linear brainstem filter	all	*B_L_*(*q*, γ) = 0.66−0.48*q*^−1^/1−0.82*q*^−1^
discrete linear reference filter	all	*M*(*q*, τ) = 0.18/1−0.82*q*^−1^

The performance of the second control scheme, in which a nonlinear adaptive cerebellum replaces the linear adaptive cerebellum of the first scheme, is also shown in figure 6. It learns to compensate well for the nonlinear plant, and the desired displacement response is accurately tracked over the full range of displacements, including larger peaks (figure 6*b*,*c*). This improvement is reflected in lower RMS errors (figure 6*d*: 0.019 mm). The number of nonlinear cerebellar elements required to achieve this reduction in error is approximately 5 (figure 6*e*).

Finally, the PID controller initially performed better than either adaptive scheme (figure 6*d*). As learning proceeded, the linear adaptive scheme came to perform similarly as indicated by RMS error, whereas the nonlinear scheme did slightly better.

**Figure 7.**
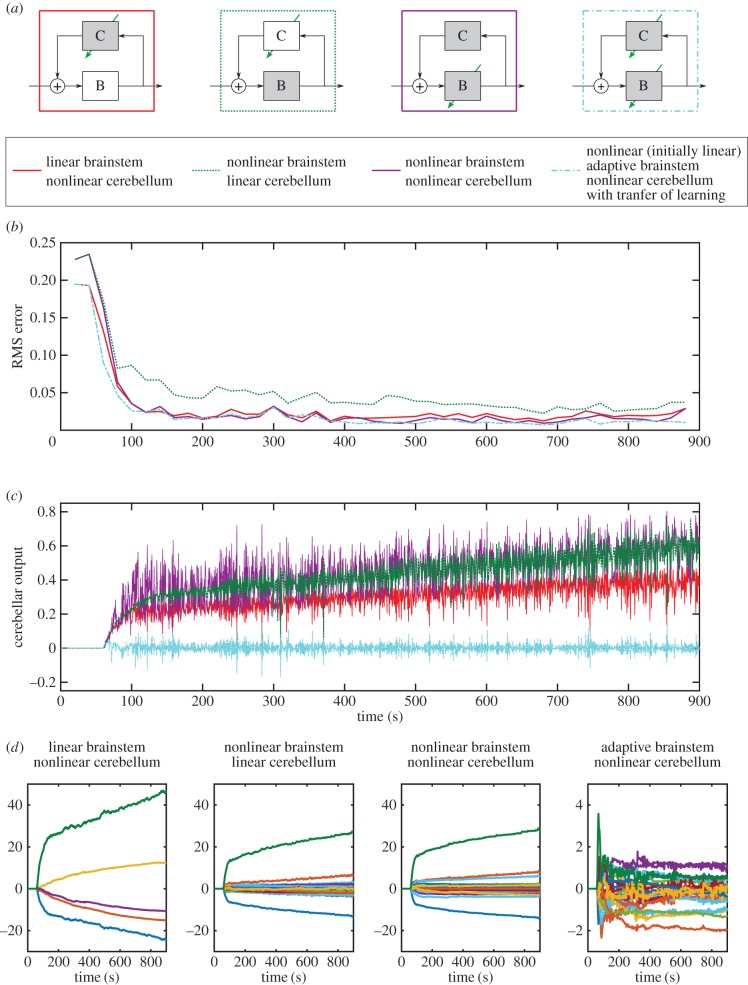
Comparison of nonlinear control strategies. Simulated results when applying different nonlinear control strategies to control of the DEA. (*a*) Diagram of the four nonlinear control schemes. An arrow indicates an adaptive element, and a shaded box a nonlinear element. Results for linear brainstem and nonlinear cerebellum (red lines) previously shown in figure 6. (*b*) Windowed RMS errors for each control scheme. (*c*) Cerebellar output for each control schemes. For the three schemes in which the brainstem is fixed, the cerebellar output increases over time, as the properties of the DEA change (‘creep’). When the brainstem is adaptive because of learning transferred from the cerebellum, the cerebellar output does not increase over time. (*d*) Evolution of cerebellar weights over time for each control scheme. Note the *y*-axis scale on the plot on the right is 10× smaller than the other plots.

The fourth control scheme to be investigated used a linear adaptive cerebellum as in the first scheme, but combined it with a nonlinear brainstem intended to capture the effects of motor unit recruitment in skeletal and EOMs (figure 7*a*). Its eventual performance was slightly worse than that of the second scheme (figure 7*b*; average final RMS errors of 0.030 mm), and learning was somewhat slower.

In the fifth and sixth control schemes, both the brainstem and cerebellum were nonlinear, but whereas in the fifth scheme, the brainstem remained fixed, in the sixth it was adaptive (figure 7*a*) with learning driven by changes in cerebellar output, as can occur in VOR adaptation. Both schemes produced good learning (steady-state RMS errors 0.015 and 0.011 mm, respectively), a value for the sixth scheme that matches the steady-state RMS errors when controlling the DEA over a reduced linear range, using a linear control scheme. In addition, the fifth scheme's method of achieving this level of performance was different. Figure 7*c* shows how cerebellar output varies over time for each of the three nonlinear schemes. If there is no transfer of learning between cerebellum and brainstem (schemes two to four), then this output gradually increases to cope with the slow ‘creep’ of plant properties (figure 1*c*). Such continual increase is undesirable, especially when the cerebellum is connected in a recurrent loop, so that large cerebellar outputs are effectively large gains in a feedback loop and can thus cause instabilities. However, when a nonlinear adaptive brainstem element is used and learning is transferred from the cerebellum to the brainstem the cerebellum output no longer increases continually over time (figure 7*c*). These differences between the control schemes are also reflected in the evolution of cerebellar weights as learning proceeds (figure 7*d*). In particular, weight change is very much reduced and stabilized when transfer to the brainstem is allowed (figure 7*d*, right-most panel).

**Figure 8.**
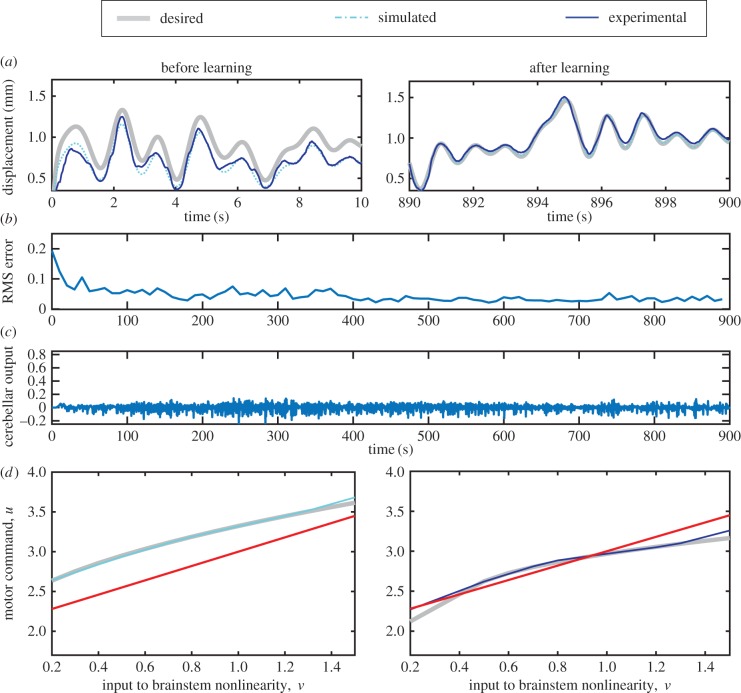
Experimental control. Experimental control when using a nonlinear cerebellum and nonlinear brainstem with transfer of learning. (*a*) Tracking a desired displacement signal in simulation and a real-time experiment. The left-hand panel shows desired and actual responses before learning and the right-hand panel the responses after learning. The simulation characterizes the actual system well. (*b*) Windowed RMS errors from real-time control experiment. (*c*) Cerebellar output during real-time control experiment. (*d*) Learnt brainstem nonlinearity in simulation (left) and experiment (right) compared with initial linear brainstem approximation. The learnt brainstem nonlinearity reasonably approximates the estimated inverse of the plant nonlinearity over the majority of input signals.

Finally, the sixth control scheme was applied to displacement control of the real-world DEA system, and the resulting performance compared with that seen in the simulation (figure 8*a*). After learning, both the simulated and real-world systems track the desired displacement response accurately. It appears that the model of the DEA used in the simulations provides a reasonable description of its dynamics, and that the control algorithm works as expected on a real-world system. RMS error is shown in figure 8*b*, and cerebellar output in figure 8*c*.

The learnt brainstem nonlinearity (from an initially linear estimate) was compared with the estimated inverse of the plant nonlinearity for both the simulated and real-world systems (figure 8*d*). The specific form of the plant nonlinearity differs between the real-world and simulated systems owing to variations in the characteristics of individual actuators [8], though the general form of the nonlinearity is similar. In both simulated and the real-world systems, the learnt brainstem nonlinearity reasonably approximates the inverse of the plant nonlinearity (for ideal compensation, the two should be equal). The approximation is less good for large and small displacements, probably because there are fewer data available to learn over these ranges.

For the results shown in figure 8, the transfer of learning from the cerebellum to brainstem was calculated using a learning rule in which previous gains are taken into account (equation (A 10) in appendix A) to provide some decorrelation of the signals being weighted. A simpler learning rule that does not include the effect of previous gains was also tested on the simulated system and gave very similar performance to that shown in figure 8 (results not shown).

## 4. Discussion

These results show that a bioinspired control scheme, based on cerebellar calibration of the VOR, is capable of compensating for the plant nonlinearities of a DEA-based actuator. Good performance was obtained with either an adaptive (cerebellar) filter using nonlinear basis functions, or a fixed brainstem nonlinearity based on recruitment of EOM. In addition, a biologically based arrangement, in which the adaptive filter teaches the brainstem model of the inverse plant, allowed the amplitude of cerebellar output to remain relatively stationary even though plant properties gradually changed with time.

We consider the implications of these findings first for EAP control, then for understanding biological control. Finally, we discuss possibilities for future work.

### 4.1. Electroactive polymer control

A wide variety of control schemes have been proposed for both ionic and dielectric EAs [9,34–40] and, at present, there appears to be no consensus about which of them is most suitable.

The schemes particularly relevant to this study are those involving inverse control. Some use non-adaptive methods, deriving a plant model by system identification techniques then inverting it (with appropriate safeguards) [34,36,37,39]. Of the studies that do involve adaptive methods, Hao & Li [35] use on online LMS algorithm to identify hysteresis parameters online, and a separate offline identification algorithm to obtain creep parameters. Sarban & Jones [38] derive a physical-based electromechanical model of the DEA, and estimate values for its 14 parameters. Druitt & Alici [9] argue that the problems of explicit modelling can be avoided by using intelligent controllers such as those based on fuzzy logic or neural networks, and demonstrate the utility of a neurofuzzy adaptive neural fuzzy inference system.

Our approach also seeks to reduce the need for offline system identification by using only a relatively crude inverse model in the ‘brainstem’, and in addition employs an adaptive filter as the intelligent part of the control system rather than a complex adaptive neural fuzzy inference system. Moreover, the brainstem model can be taught, which both reduces dependence on *a priori* estimates, and is also particularly suitable for tracking slow changes in performance (‘creep’) without long-term increases in adaptive-controller output. Finally, the basic structure of the control scheme suggests immediate possibilities for compensating for temperature effects or poor manufacturing tolerances, for implementing impedance control in agonist–antagonist EAPs, and for augmenting feedback in mixed feedback–feedforward control schemes (discussed further in §4.3.).

### 4.2. Biological control

The importance of using robots to test hypotheses about neural function is well recognized [24,41], and previous work has explored how cerebellar-inspired control schemes could be applied to robots [42–45]. The success of the adaptive-filter model embedded in the recurrent architecture in controlling DEAs in their linear range [7] prompted its extension here to the nonlinear range. The results have three implications for understanding neural function.

The first concerns the adaptive filter model of the cerebellar microcircuit. How granular layer processing could generate the equivalent of basis filters is not well understood, although current approaches using insights from reservoir computing are attracting interest [46,47]. These treat the granular layer as a recurrent inhibitory network, in which granule cells project to inhibitory Golgi cells which, in turn, project back to the synapses between mossy fibres and granule cells (figure 2*a*). If the recurrent inhibition is allowed to change rapidly, then the resultant dynamics are very rich and can generate a wide variety of basis functions [47]. However, some of the Golgi cell inhibition appears to change very slowly, which has led to the suggestion that the granular layer generates piecewise linear approximations of nonlinear functions [19]. The present results indicate that such basis functions can be used, in practice, to compensate for certain kinds of nonlinear plant.

Second, it appears that a distributed representation of the approximate inverse model in the brainstem [12] can also help to compensate for the same kind of nonlinearity. In the oculomotor system, the agonist force needed to maintain eccentric eye-position increases supralinearly with position, yet the firing rate of individual ocular motoneurons (OMNs) varies linearly with position. However, OMN thresholds (and slopes) vary over a wide range. It has been proposed that such recruitment can help to linearize the oculomotor plant (references in [48]). Results here suggest that this putative mechanism can work in practice.

Finally, the results indicate that transferring learning from cerebellum to brainstem allows the system to compensate for creep with little increase in cerebellar output (figure 7*c*). In the case of VOR adaptation, where there is good evidence that in particular circumstances a similar transfer occurs [32], modelling indicates that the brainstem can learn new values of VOR gain that allow the system to operate at high frequencies (up to 25 Hz) despite a substantially delayed retinal-slip error signal (approx. 100 ms) [23]. The results here suggest learning transfer may have more generic benefits in stabilizing adaptive control output by ensuring large cerebellar outputs do not affect the stability of the recurrent loop. They provide further computational evidence as to why a powerful computational device such as the adaptive filter model of the cerebellum requires an additional site of plasticity and agree with previous computational predictions that learning occurs first in the cerebellar cortex, before transferring to the brainstem [23].

### 4.3. Future work

We need to understand how to control DEAs arranged in agonist–antagonist pairs [3,49]. Analysis of the oculomotor system suggests that small changes in conjugate eye-position in the horizontal plane are maintained by the minimum possible change in motor commands (the minimum-norm rule) [22]. It is therefore possible that the control scheme investigated here, which is based on the oculomotor system, could be extended to the optimal control of agonist–antagonist DEA pairs. If so it could be applied generally, and would be of special relevance to the use of EAPs as neuroprostheses [50,51] and as eye muscles for an android robot [52].

## Supplementary Material

Data_Cerebellar_Inpsired_Adaptive_Control_for_Nonlinear_Artificial_Muscle

## Appendix A. Details of control algorithms

The control algorithms are described here using discrete time notation, where *k* denotes the time step. Filters are described in discrete time using the notation *D*(*q*, γ), where *D*(*q*, γ) is a linear discrete time filter, *q* the shift operator () and *γ* a vector of filter parameters.

**A.1. Linear control**

The plant being controlled is described as

A 1

where , *x_k_* is the measured output, *u_k_* the measured input, *n* the system order and *f_o_* a continuous nonlinear function. We assume that there exists a unique, continuous function inverse , such that

A 2

where  is the inverse mapping of *f_o_* and describes a one-to-one mapping from *x* → *u*.

The cerebellar element **C** in figure 3*b* is modelled as an adaptive filter (figure 2), where the output (*z_k_*) is given as a weighted sum of filtered and optimized input signals. Thus, for time step *k*

A 3

where  *w_i,k_* denotes the *i*th weight at time step *k*, and  *p_i,k_* denotes the *i*th parallel fibre at time step *k*. These weights are adjusted by the error signal *e* (corresponding to climbing fibre input) according to the LMS learning rule [25].

A 4

where  ***p****_k_* denotes the parallel fibre signals being filtered through reference model filter (see table 2 for the discrete time reference filter definition), and ***e****_k_* is the sensory error signal, or difference between desired and actual system output .

In the present model, the basis functions implemented by the filters *G_1_* … *G_N_* are alpha functions (second-order low pass filters with a repeated root), described by a single parameter , where *T_i_* is the time constant of the *i*th fixed filter (see table 2 for the discrete time alpha filter approximation). These basis functions replace the most commonly used tapped delay line FIR filter and greatly reduce the number of adaptable weights required [53,54]. The output of these filters is denoted ***g****_k_*. To speed learning, the outputs of these filters ***g****_k_* are transformed by the fixed matrix *Q* to give parallel fibre signals ***p****_k_*

A 5

where  and is designed offline to exactly orthonormalize the brainstem output when there is no cerebellar contribution, i.e. *z_k_* = 0 (for further details on the design of *Q*, see [7]).

**A.2. Nonlinear control-adaptive filter**

In the nonlinear adaptive filter, the signals being weighted are nonlinear functions of the input signal, and the output is a linear-in-weights combination of these signals. For the linear case, the vector ***g****_k_* is the output of a bank of fixed, linear filters (figure 3*b*). Here, we extend this to nonlinear case (figure 4*d*) and express ***g****_k_* as

A 6

where *f*_1_ is a nonlinear function of filter outputs, and *f*_2_ is a nonlinear function of filter inputs, *n*_f_ is the number of filters and  is a fixed discrete time filter, where *γ* is vector of filter parameters and we call the bank of fixed filters ‘basis functions’,  is a discrete bias term. For the case  and  equation (A 6) reduces to a linear adaptive filter. Here, we do not transform the filter outputs, so trivially . We construct nonlinear basis by thresholding inputs to the linear basis filters such that only motor commands above a certain threshold are input—a range of threshold values as well as the original motor command signal were used (inspired by the suggestion that the granular layer generates threshold-linear processing elements). This nonlinear transformation of inputs can be expressed as

A 7

The input *u_k_* is transformed into a vector that contains *u_k_* as well as thresholded versions of *u_k_*.  is the heaviside step function, *η* is the number of thresholded terms and  is a vector of threshold cut of values. Equation (A 7) can be described compactly as , where ***q****_k_* is a vector of thresholded signals.

**A.3. Nonlinear control-brainstem**

Figure 4*a* shows a general Hammerstein model of a plant, and figure 4*b* shows its nonlinear inverse controller which consists of an LDS (i.e. a fixed linear filter) followed by an SNL. The output *v_k_* of the fixed linear filter is given as

A 8

The SNL of the brainstem is designed to compensate for the plant nonlinearity (denoted ), assuming there exists a unique, continuous function , that gives the inverse mapping of  (see above). Perfect compensation of the nonlinearity is achieved if the SNL in the brainstem equals , and so the brainstem nonlinearity is designed to approximate . Here, we use a series of piecewise linear elements to approximate a continuous nonlinear function (as shown figure 4*e* and inspired by threshold elements found in the brainstem)

A 9

where *m* is the number of thresholded, piecewise linear terms,  a vector of threshold cut-off values and  is the gain of the *j*th piecewise linear element.

**A.4. Linear proportional-integral-derivative control**

A linear proportional-integral-derivative controller (PID controller) was also applied to the simulated DEA (see section Control evaluation in appendix). The discrete time PID controller is

A 10

where *K_p_*, *K_i_*, *K_d_* are the controller gains, *T_d_* a term used to limit the high-frequency gain of the controller and *T*_s_ the sampling period (0.02). The controller parameters (*K_p_* = 1.3, *K_i_* = 3, *K_d_* = 5.3, *T_d_* = 4.7) were estimated as the parameters that minimized the total squared errors over time when controlling the simulated DEA.

**A.5. Learning in the brainstem**

The gains of the piecewise linear elements can be learnt online, by transferring learning from the cerebellum back to the brainstem. This is done using a Hebbian learning rule, where the gain of the *j*th piecewise linear element at time step *k* + 1 for  is given as

A 11

where *ζ* is the learning rate and  represents the *j*th piecewise linear element at time *k*, i.e. . The additional term at the end of the expression for cases when  removes the effect of changes in gains at lower thresholds on the gain at higher thresholds.

**A.6. Parameters**

The algorithm requires the following parameters to be specified parameters before implementation: rate of error learning (*β*); rate of brainstem learning (*ζ*); linear brainstem filter (); time constant of reference model filter (*τ*); number of thresholded terms in the cerebellum (*η*) and the corresponding cut-off values (); number of alpha filters (*n_f_*), and corresponding time constants (); number of piecewise linear terms in the brainstem (*m*), and corresponding cut-off values (); scale of cerebellar bias ().

Some parameters differed between particular control conditions, whereas others were fixed for all experiments. Parameter values and the initial conditions for each control condition are described in Control evaluation section.

**A.7. Control evaluation**

The control algorithm was implemented both online in the real system (as described above), and in simulation. In simulation, a previously identified model of the DEA plant was used instead of the physical DEA (details of the model and parameter estimation are provided in [7]). The plant model used to transform an input *u_k_* into an output *x_k_* is described in equations (A 12)–(A 14) (see also figure 4*a*).

A 12

A 13

A 14

The model parameters (*b_k_* = 0.3, *c_k_* = −0.4, *d_k_* = 0.5, *e_k_* = 2.2) were set to produce similar behaviour to the actual actuator, and adapted each time step (by *δ_b_* = 7 × 10^−8^, *δ_c_* = 7 × 10^−6^, *δ_d_* = 1.3 × 10^−6^, *δ_e_* = 2.3 × 10^−6^).

The control algorithm was tested under different conditions by varying the control parameters. The following conditions were tested: linear control with a linear brainstem and linear cerebellum (first scheme); nonlinear control with a linear brainstem and nonlinear cerebellum (second scheme); a PID-based linear controller (third scheme); nonlinear control with a fixed brainstem nonlinearity and linear cerebellum (fourth scheme); nonlinear control with a fixed brainstem nonlinearity and nonlinear cerebellum (fifth scheme); nonlinear control using a nonlinear brainstem with adaptive piecewise linear gains and a nonlinear cerebellum (sixth scheme); all conditions were tested in simulation, and the first and last were also tested on the physical actuator.

Details of the parameters and initial conditions for each experimental case are provided in table 2. In each control experiment, the reference signal *r_k_* was low-pass filtered white noise with frequency range 0–1 Hz.
